# The structure, dynamics and selectivity profile of a Na_V_1.7 potency-optimised huwentoxin-IV variant

**DOI:** 10.1371/journal.pone.0173551

**Published:** 2017-03-16

**Authors:** Sassan Rahnama, Jennifer R. Deuis, Fernanda C. Cardoso, Venkatraman Ramanujam, Richard J. Lewis, Lachlan D. Rash, Glenn F. King, Irina Vetter, Mehdi Mobli

**Affiliations:** 1 Centre for Advanced Imaging, The University of Queensland, St Lucia, QLD, Australia; 2 Institute for Molecular Bioscience, The University of Queensland, St Lucia, QLD, Australia; 3 School of Biomedical Sciences, The University of Queensland, St Lucia, QLD, Australia; Universitatsklinikum Wurzburg, GERMANY

## Abstract

Venom-derived peptides have attracted much attention as potential lead molecules for pharmaceutical development. A well-known example is Huwentoxin-IV (HwTx-IV), a peptide toxin isolated from the venom of the Chinese bird-eating spider *Haplopelma schmitdi*. HwTx-IV was identified as a potent blocker of a human voltage-gated sodium channel (hNa_V_1.7), which is a genetically validated analgesic target. The peptide was promising as it showed high potency at Na_V_1.7 (IC_50_ ~26 nM) and selectivity over the cardiac Na_V_ subtype (Na_V_1.5). Mutagenesis studies aimed at optimising the potency of the peptide resulted in the development of a triple-mutant of HwTx-IV (E1G, E4G, Y33W, m_3_-HwTx-IV) with significantly increased potency against hNa_V_1.7 (IC_50_ = 0.4 ± 0.1 nM) without increased potency against hNa_V_1.5. The activity of m_3_-HwTx-IV against other Na_V_ subtypes was, however, not investigated. Similarly, the structure of the mutant peptide was not characterised, limiting the interpretation of the observed increase in potency. In this study we produced isotope-labelled recombinant m_3_-HwTx-IV in *E*. *coli*, which enabled us to characterise the atomic-resolution structure and dynamics of the peptide by NMR spectroscopy. The results show that the structure of the peptide is not perturbed by the mutations, whilst the relaxation studies reveal that residues in the active site of the peptide undergo conformational exchange. Additionally, the Na_V_ subtype selectivity of the recombinant peptide was characterised, revealing potent inhibition of neuronal Na_V_ subtypes 1.1, 1.2, 1.3, 1.6 and 1.7. In parallel to the *in vitro* studies, we investigated Na_V_1.7 target engagement of the peptide *in vivo* using a rodent pain model, where m_3_-HwTx-IV dose-dependently suppressed spontaneous pain induced by the Na_V_1.7 activator OD1. Thus, our results provide further insight into the structure and dynamics of this class of peptides that may prove useful in guiding the development of inhibitors with improved selectivity for analgesic Na_V_ subtypes.

## Introduction

Voltage-gated ion channels are ubiquitous signalling molecules that respond to changes in the cell membrane polarisation caused by ion concentration gradients between the intra- and extracellular environment [1]. They are particularly important in the initiation and propagation of electrical signals in excitable cells. Dysfunction of these transmembrane proteins can lead to a range of severe channelopathies including epilepsy, cardiac arrhythmias and various pain disorders. Their central role in these pathologies makes them attractive pharmaceutical targets [2].

One of the most attractive drug targets to emerge in recent years in the ion-channel field is the Na_V_1.7 subtype of voltage-gated sodium channels (Na_V_s). Mutations in *SCN9A*, the gene encoding Na_V_1.7, which lead to a loss of function, result in congenital insensitivity to pain (CIP) with anosmia as the only other sensory deficit, whilst gain-of-function mutations are associated with extreme sensitivity to pain (erythromelalgia and paroxysmal extreme pain disorder) [3, 4]. Thus, inhibitors of Na_V_1.7 are promising analgesic leads, and indeed, local anasthetics such as lidocaine are known to non-selectively inhibit Na_V_ channels [2]. Unfortunately, off-target effects at other homologous Na_V_s (1.1, 1.2, 1.4, 1.5 and 1.6) are likely to lead to severe side-effects including seizures, arrhythmias and impaired motor function [5].

Small disulfide-rich peptides isolated from the venom of animals are natural modulators of voltage-gated ion channels. The larger footprint of these peptides, compared to small molecule drugs, is thought to provide an increased opportunity for development of more selective ligands [6]. Peptides are therefore considered a goldilocks compromise between small molecules and antibodies, with the former providing limited selectivity and the latter being associated with high production costs. Thus, there are currently significant efforts in industry and academia to discover novel peptides as lead molecules for development of biologics that target ion channels [6–14].

Perhaps the best-studied venom peptide inhibitor of Na_V_1.7 is the 35-residue HwTx-IV, isolated from the venom of the Chinese bird-eating spider *Haplopelma schmitdi* [15]. The peptide completely inhibits Na_V_1.7 with an IC_50_ in the low nanomolar range (~26 nM) [16]. Detailed characterisation of the toxin-channel interaction revealed that the peptide binds to one of the four voltage sensor domains (VSD) of the channel [16, 17]. This is in contrast to extant small molecule drugs that bind to the central pore region, such as local anaesthetics [18]. The channel pore is more highly conserved among the different Na_V_ subtypes compared to the VSD, making the VSD an attractive target for development of selective drugs, and small molecules that target the VSD are being developed [7, 11].

Na_V_s contain four homologous but non-identical VSDs that control the gating of the channel. Each VSD consists of four transmembrane helical segments (S1-S4) connected via intra- and extra-cellular loops. VSDs of domains II and IV (VSD_II_ and VSD_IV_) have been shown to be attractive targets as they control channel opening and inactivation, respectively. Elegant work by Xiao *et al*. showed that HwTx-IV specifically binds to acidic residues in the S1-S2 and S3-S4 loops of VSD_II_; rat Na_V_1.2, Na_V_1.3 and human Na_V_1.7, all of which contain E818 in S3-S4, are sensitive to the toxin whilst Na_V_1.4 and Na_V_1.5, which lack an acidic residue in this position, are insensitive to HwTx-IV. Subsequent work by others defining the pharmacophore of the peptide through extensive mutagenesis identified that in particular residues W30 and K32 are required for Na_V_ activity, whilst mutations in the N and C terminal parts of the peptide were found to improve binding to Na_V_1.7 without improvement in activity against Na_V_1.5 [12, 14]. This led to the development of a triple mutant of HwTx-IV (E1G, E4G, Y33W HwTx-IV, hereafter m_3_-HwTx-IV), which is one of the most potent blockers of Na_V_1.7 reported to date (IC_50_ ~ 0.5 nM) [14]. Despite the extensive work carried out on HwTx-IV, its complete subtype selectivity is not known. Similarly, although the structure of the wild type (WT) peptide and several variants have been determined [19, 20], the effect of the triple mutation on the pharmacophore is not known and a high quality structure would provide valuable information for rationalising the observed gain in potency.

To this end, we produced a recombinant, isotopically labelled version of m_3_-HwTx-IV, which allowed us to determine (1) a high-resolution 3D structure of the peptide using heteronuclear NMR and (2) the dynamics of the peptide using ^15^N relaxation experiments. Although the overall fold of m_3_-HwTx-IV remained the same as the parent toxin [14], our dynamics data show large amplitude motion in the pharmacophore region indicating that key residues undergo conformational exchange in the μs-ms time scale. *In vitro* and *in vivo* functional data indicate that m_3_-HwTx-IV has the same Na_V_ selectivity profile as the WT toxin, and we show for the first time that the peptide is a potent inhibitor of Na_V_1.6 and Na_V_1.1 channels. The full selectivity profile of the channel reveals that Na_V_ isoforms 1.1, 1.2, 1.3, 1.6 and 1.7 are sensitive to the toxin whilst isoforms 1.4, 1.5 and 1.8 are not. Finally, we show that the peptide has Na_V_1.7 on-target activity *in vivo* using a recently developed Na_V_1.7 target-engagement mouse model [21]. Our findings provide further insight into the structure, function and activity of this fascinating class of peptides in the pursuit of turning venoms into analgesic drugs.

## Materials and methods

### Recombinant peptide production

A codon-optimised synthetic gene encoding m_3_-HwTx-IV was synthesised by GeneArt (Thermo Fisher Scientific) and subcloned into an in-house, modified version of pLIC expression vector, between the *Kpn*I and *Sac*I restriction sites, in frame with a MalE signal sequence to direct secretion of the fusion-protein to the periplasmic space of *E*. *coli*, where disulfide-bond formation and folding of the molecule takes place [22]. Competent *E*. *coli* BL21 (DE3) cells were transformed with the plasmid encoding His_6_-MBP-m_3_-HwTx-IV fusion protein. Cells were grown on a LB agar plate supplemented with 100 μg/mL ampicillin (Amp). LB/Amp (100 μg/mL) medium was inoculated from a single colony or glycerol stock and the cells were grown for ~18 h (overnight) at 30°C whilst shaking at 120 rpm until reaching an OD_600_ of ~2. Fresh LB/Amp solution was inoculated with the pre-culture to an OD_600_ of ~0.05 under agitation at 120 rpm in 5 litre baffled flasks at 37°C until reaching an OD_600_ of ~0.4, at which time the growth temperature was reduced to 16°C. Protein production was induced at OD_600_ of ~0.8 by addition of isopropyl β-D-1-thiogalactopyranoside (IPTG) to a final concentration of 0.2 mM and growth was continued overnight at 16°C. The cells were harvested by centrifugation (6,000 *g* for 15 min at 4°C) and the cell pellets stored at –80°C for subsequent purification. In order to produce uniformly ^13^C/^15^N-labelled m_3_-HwTx-IV peptide, LB broth was replaced with M9 minimal medium supplemented with ^15^NH_4_Cl and ^13^C_6_-glucose as the sole nitrogen and carbon sources, respectively [23].

### Peptide purification

Cells overexpressing His_6_-MBP-m_3_-HwTx-IV fusion protein were resuspended by stirring in lysis buffer (40 mM Tris-HCl, pH 8.0, and 400 mM NaCl) at 4°C for 1 h, or until reaching complete homogeneity. Cells were lysed by continuous flow cell disruption (Constant Systems Ltd., TS Series Benchtop; triple passage at constant pressure of 32 kpsi). The cell lysate was centrifuged (40,000 *g*, 30 min, 4°C) to remove cell debris. The supernatant was loaded onto a Ni-NTA column equilibrated with lysis buffer. The column was washed with 3–5 column volumes (CV) of lysis buffer, then non-specifically bound proteins were eluted with the same buffer containing 40 mM imidazole. The fusion protein was then eluted from the column with lysis buffer supplemented with 250 mM imidazole. The peak fractions were pooled and then desalted and concentrated using an Amicon Ultra-15 centrifugal filter unit (Merck Millipore) with a 30 kDa cut-off membrane. The protein was then digested to yield His_6_-MBP and m_3_-HwTx-IV peptide using 50 μg/ml tobacco etch virus (TEV) protease in a freshly prepared redox buffer containing 3 mM reduced glutathione (GSH) and 0.3 mM oxidised glutathione (GSSG), gently shaking overnight, at room temperature. Fusion protein cleavage was confirmed by SDS-PAGE, then the cleavage reaction mixture was acidified by adding 1% trifluoroacetic acid (TFA). Precipitated protein was removed by centrifugation followed by membrane filtration (0.45 μm). Reverse-phase (RP) HPLC purification of m_3_-HwTx-IV was performed using a C_4_ semi-preparative column (Phenomenex). The bound peptide was eluted at 3 ml/min with a linear gradient of 5–80% solvent B (90% Acetonitrile (ACN), 0.043% TFA) in solvent A (0.05% TFA in water). UV absorbance was monitored at 214 and 280 nm and fractions corresponding to the peptide of interest were pooled, lyophilised and resuspended in pure water. UV absorbance was measured (Thermo Scientific™ NanoDrop 2000) at 280 nm, and the concentration was calculated using the predicted extinction coefficient of the peptide. Identity and purity of the peptide was further characterised by liquid chromatography mass spectrometry (LC-MS), matrix-assisted laser desorption/ionisation time-of-flight (MALDI-TOF) mass spectrometry and analytical RP-HPLC with a C_18_ analytical column (Phenomenex) using a gradient of 1% solvent B/min at a flow rate of 1 ml/min.

### Mass spectrometry

MALDI-TOF MS performed on a Bruker autoflex speed High-Performance MALDI-TOF System (Bruker, MA, USA) was employed to determine peptide mass. RP-HPLC fractions were mixed (2:1 v/v) with α-cyano-4-hydroxycinnamic acid (CHCA) matrix (5 mg/ml in 50/50 ACN/H_2_O) on a MALDI plate spot and dried at room temperature. The MALDI-TOF mass spectrum was acquired in positive reflector mode. Applied Biosystems Data Explorer™ was used for analysis of monoisotopic [M + H]^+^ ions MS data.

### NMR structure determination

All NMR data were recorded using a 300 μl samples, added into a susceptibility-matched 5 mm micro tube (Shigemi Inc. Japan), prepared by dissolving ^15^N or ^15^N/^13^C-labeled m_3_-HwTx-IV to a final concentration of 400 μM in 20 mM sodium acetate solution, pH 5 with 5% (v/v) D_2_O. All spectra were acquired at 25 ^o^C using a 900 MHz Bruker Avance NMR spectrometer equipped with a cryogenically cooled probe (cryoprobe). Resonance assignments were obtained using two-dimentional (2D) ^1^H-^15^N heteronuclear single quantum coherence (HSQC), 2D ^1^H-^13^C-HSQC, 3D CBCA(CO)NH, 3D HNCACB, 3D HNCO, 3D HBHA(CO)NH and four-dimensional (4D) HCC(CO)NH-TOCSY spectra. 3D and 4D spectra were acquired using non-uniform sampling [24, 25] and processed using the Rowland NMR Toolkit (http://rnmrtk.uchc.edu/rnmrtk/RNMRTK.html). Maximum entropy parameters were selected automatically as described previously [26]. 3D ^15^N and ^13^C (separate spectra for aliphatic and aromatic regions) filtered NOESY-HSQC spectra (200 ms mixing time) were acquired using linear sampling and processed using the Rowland NMR Toolkit. Data were analysed in CcpNmr Analysis v2.4 (University of Cambridge, UK) [27]. NOESY cross peaks were manually picked and supplemented with dihedral angles derived from chemical shifts using the TALOS software [28], and used for structure calculation using CYANA v3.0. [29] The NOEs were automatically assigned by CYANA. 200 structures were calculated and an ensemble of 20 structures with the lowest CYANA target function were selected.

### Dynamics of the peptide

#### NMR spin relaxation

NMR experiments for measuring longitudinal (R1) and transverse relaxation rates (R2) and ^1^H-^15^N steady-state NOEs were acquired using a single-scan interleaved pulse sequence on a 700 MHz Bruker spectrometer equipped with a *z*-gradient cryoprobe. Data were acquired at 25°C using a 200 μM sample of ^15^N labeled m_3_-HwTx-IV. R1 spectra were recorded with 7 delay times (10*, 50, 300*, 500, 700, 900*, 1100 ms) as were R2 spectra (16*, 32, 64, 96*, 128, 160, 192 ms), where delays marked with an asterisk were recorded twice to confirm data reproducibility. Steady-state ^1^H-^15^N heteronuclear NOEs were acquired with a relaxation delay of 9 s. Spectra were processed using the Rowland NMR toolkit and analysed using CcpNMR [27].

### hNa_V_1 activity using patch-clamp electrophysiology

Patch-clamp experiments used HEK293 cells expressing either of hNa_V_1.1 –hNa_V_1.5 and the β1 auxiliary subunit (SB Drug Discovery, Glasgow, UK) or Chinese Hamster Ovary (CHO) cells expressing hNa_V_1.6, hNa_V_1.7 and hNa_V_1.8/β3 (ChanTest Corp). Na^+^ currents were measured by automated whole-cell patch clamp (QPatch 16X; Biolin Scientific A/S, Ballerup, Denmark). The extracellular solution comprised (in mM) 1 CaCl_2_, 1 MgCl_2_, 5 HEPES, 3 KCl, 140 NaCl, 0.1 CdCl_2_ and 20 TEA-Cl at pH 7.3 and 320 mOsm, and the intracellular solution comprised (in mM) 140 CsF, 1/5 EGTA/CsOH, 10 HEPES and 10 NaCl at pH 7.3 and 320 mOsm. The elicited currents were sampled at 25 kHz and filtered at 4 kHz. Cells were maintained at a holding potential –80 mV and Na^+^ currents elicited by 20 ms voltage steps to 0 mV (+10 mV for Na_V_1.8) from a –120 mV conditioning pulse applied for 200 ms. To obtain concentration–response curves, cells were incubated for 5 min with increasing concentrations of toxin. All experimental data was analysed using QPatch Assay Software v5.0 (Biolin Scientific A/S, Ballerup, Denmark).

### hNa_V_1 activity using FLIPR membrane potential assay

Human embryonic kidney (HEK) 293 cells heterologously expressing human Na_V_1.1–1.8 (SB Drug Discovery) were cultured in minimal essential medium (MEM) containing 10% v/v fetal bovine serum (FBS) and selection antibiotics as recommended by the manufacturer. Cells were grown in a humidified 5% CO_2_ incubator at 37 °C, grown to 70–80% confluence, and passaged every 3–4 days using TrypLE Express (Invitrogen). Cells were plated in 384-well clear-bottom black imaging plates (Corning, NY, USA) at a density of 10,000 to 15,000 cells per well. After 48 h, cells were loaded with (20 μl per well) red membrane potential dye (Molecular Devices, Sunnyvale CA, USA) diluted in physiological salt solution [PSS; composition (in mM) 140 NaCl, 11.5 glucose, 5.9 KCl, 1.4 MgCl_2_, 1.2 NaH_2_PO_4_, 5 NaHCO_3_, 1.8 CaCl_2_ and 10 HEPES pH 7.4] and incubated for 30 min at 37°C in a humidified 5% CO_2_ incubator. m_3_-HwTx-IV (10 nM– 30 μM) was diluted in PSS/0.1% bovine serum albumin (BSA) and pre-incubated for 5 min before stimulating Na_V_1.1–1.7 using veratridine (60 μM) or Na_V_1.8 using deltamethrin (150 μM). Changes in membrane potential were assessed using a fluorescence imaging plate reader (FLIPR^TETRA^, Molecular Devices) (excitation 515 to 545 nm, emission 565 to 625 nm) every second for 300 s after adding agonists.

### *In vivo* activity assay

#### Animals

Adult male C57BL/6J mice aged 6–8 weeks were used for behavioural assessments. Mice were housed in groups of 2–4 per cage, under 12 h light-dark cycles with standard rodent chow and water *ad libitum*. Ethical approval for *in vivo* animal experiments was obtained from the University of Queensland animal ethics committee. Experiments involving animals were conducted in accordance with the Animal Care and Protection Regulation Qld (2012), the *Australian Code of Practice for the Care and Use of Animals for Scientific Purposes*, 8th edition (2013) and the *International Association for the Study of Pain Guidelines for the Use of Animals in Research*.

#### OD1 model

The Na_V_1.7 activator OD1 (300 nM) was co-administered alone (control; n = 5) or with m_3_-HwTx-IV at a concentration of 100 nM (n = 4) or 300 nM (n = 3) diluted in saline/0.1% BSA by intraplantar (i.pl.) injection in a volume of 40 μL under light isoflurane (3%) anesthesia in mice as previously described [21]. Mice were then individually placed in polyvinyl boxes (10 x 10 x 10 cm) and allowed to recover from anesthesia. Immediately upon recovery, spontaneous pain behaviours (number of paw lifts, licks, shakes, and flinches) were recorded on video, and later counted by an investigator, unaware of the treatment each individual animal received, for 40 min post-injection at 5-min intervals. Data were plotted and analysed using GraphPad Prism. Statistical significance was defined *P < 0.05 and was determined using one-way ANOVA with Dunnett’s post-test compared to control.

## Results

### Production of recombinant m_3_-HwTx-IV

**Fig 1:**
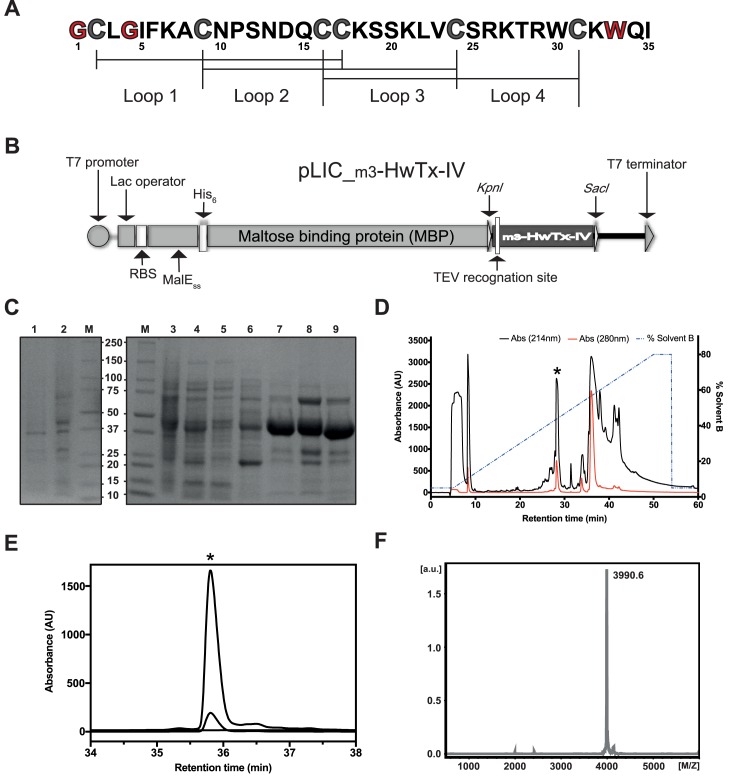
Recombinant production of m_3_-HwTx-IV. **(A)** Primary structure of m_3_-HwTx-IV. Mutated residues relative to the native toxin are highlighted in red. Disulfide bridge connectivity is shown below the sequence. **(B)** Schematic representation of the pLIC vector used for periplasmic expression of m_3_-HwTx-IV. The coding region includes a MalE signal sequence (MalE_SS_) for periplasmic export, a His_6_ affinity tag for a facile nickel affinity purification, a maltose binding protein (MBP) fusion tag for peptide solubility/stability enhancement, and a codon-optimised gene encoding m_3_-HwTx-IV, with a TEV protease recognition site inserted between the MBP and peptide-coding regions. The locations of ribosome-binding site (RBS) and other key elements of the vector are shown. **(C)** SDS-PAGE gel illustrating different steps in the purification of m_3_-HwTx-IV. Lanes are as follows: M, molecular weight markers (Bio-Rad); lane 1, *E*. *coli* cell extract prior to IPTG induction; lane 2, *E*. *coli* cell extract after IPTG induction; lane 3, lysate resulting from cell disruption; lane 4, soluble cell extract; lane 5, flow through after loading cell lysate on Ni-NTA resin; lane 6, eluate from washing Ni-NTA resin with 40 mM imidazole pre-elution buffer; lane 7, eluate from washing Ni-NTA resin with 250 mM imidazole elution buffer (the His_6_-MBP-m_3_-HwTx-IV fusion protein is evident at ~43 kDa); lane 8, fusion protein prior to addition of TEV protease; lane 9, sample after TEV protease addition showing cleavage of fusion protein. **(D)** RP-HPLC chromatogram showing initial purification of m_3_-HwTx-IV using a semi-preparative C_4_ column; the peptide eluted with a retention time of ~28 min. **(E)** RP-HPLC chromatogram of m_3_-HwTx-IV obtained using an analytical C_18_ column. The asterisk denotes the peak corresponding to correctly folded recombinant m_3_-HwTx-IV. **(F)** Molecular mass of the purified recombinant peptide was confirmed by MALDI-TOF MS (obs. = 3990.6 Da; calc. = 3992.7 Da–fully oxidised), indicating formation of the expected three disulfide bonds.

An efficient method was required to provide sufficient amounts of m_3_-HwTx-IV to perform the structural and functional studies. Therefore, we created a construct that enables expression of a His_6_-MBP-m_3_-HwTx-IV fusion protein in the periplasm of *E*. *coli*, where the disulfide-bond machinery of the cell is located [22]. The fusion protein was purified from the soluble cell fraction using Ni-NTA affinity chromatography (Fig 1C). m_3_-HwTx-IV was cleaved from the His_6_-MBP fusion tag using TEV protease, then semi-preparative RP-HPLC (Fig 1D) was employed to yield a single dominant disulfide-bond isoform with a purity of >98% as assessed by analytical RP-HPLC and MALDI-TOF MS (Fig 1E). The activity of the peptide against hNa_V_1.7 was initially confirmed using a FLIPR assay, then by automated patch-clamp electrophysiology as described below. The final yield of recombinant m_3_-HwTx-IV was ~0.6 mg per litre of bacterial culture.

### Structure of m_3_-HwTx-IV

**Fig 2:**
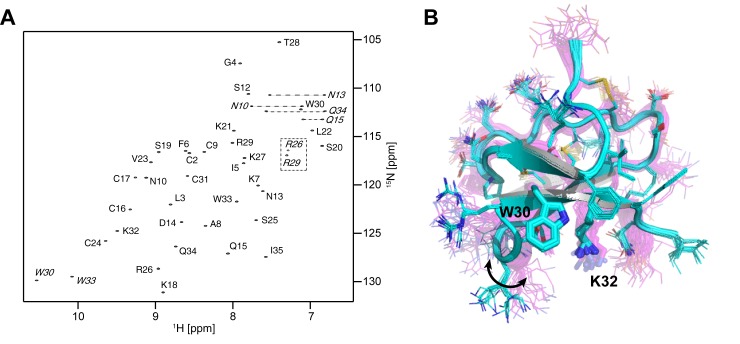
**(A**) ^**1**^**H-**^**15**^**N HSQC spectrum of m**_**3**_**-HwTx-IV**. Peaks represent backbone amide NH correlations. Sidechain correlations are annotated with italic font and the folded arginine sidechains are marked with a dotted box. NH_2_ groups are connected using a dashed line. **(B) High-resolution structure of m**_**3**_**-HwTx-IV.** Ensemble of 20 structures are shown in cyan (PDBID: 5T3M). The sidechains are shown in line representation and the critical W30/K32 residues are shown in sticks. The structure of the WT peptide (1mb6) is shown in magenta but using the same line/stick representation as for the mutant structure. The higher resolution of the new structure is evident, particularly in the definition of sidechain orientations. The arrow highlights the variation in conformation of the dynamic loop 4 between the two structures.

**Fig 3:**
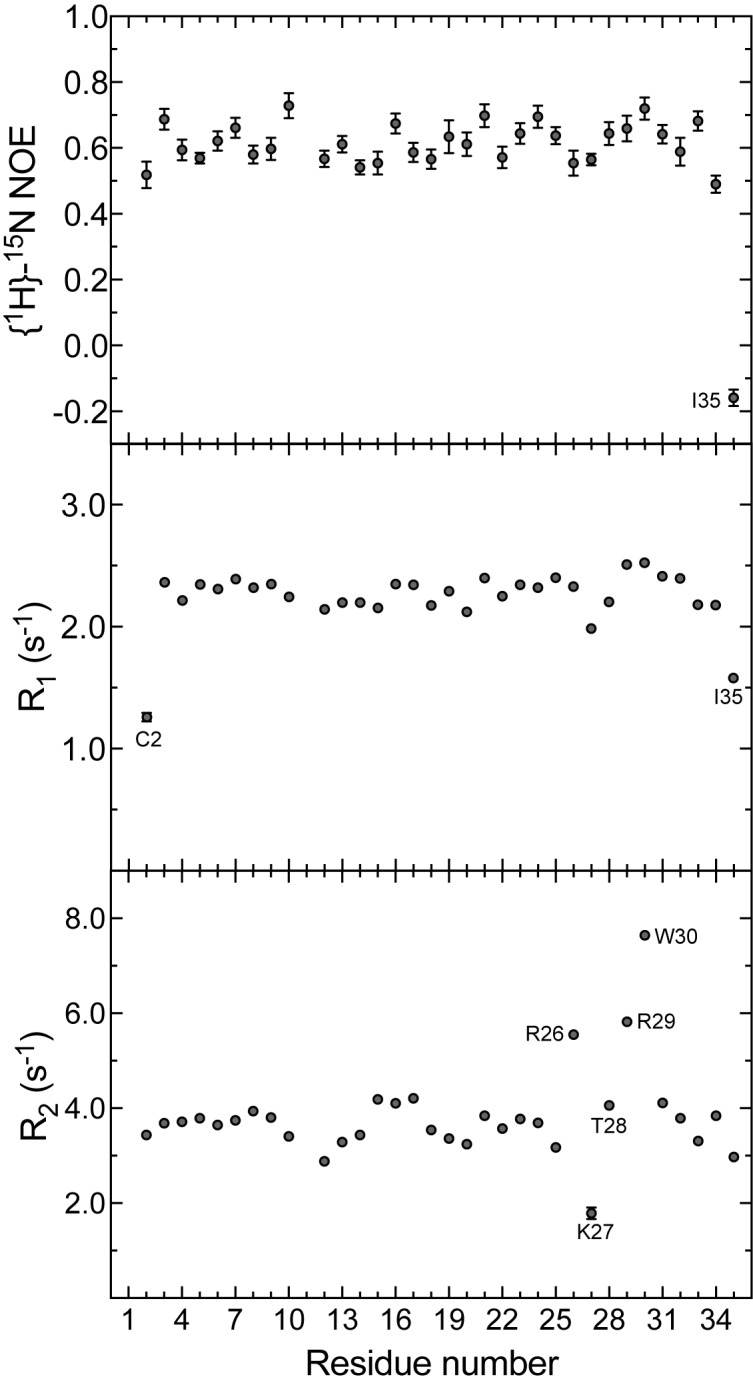
^1^H-^15^N steady state NOE and longitudinal (R1) and transverse (R2) ^15^N relaxation rates obtained for m_3_-HwTx-IV at a field-strength of 700 MHz. Data were acquired at 25˚C using a 200 μM sample of ^15^N labeled m_3_**-**HwTx-IV. The error bars in R1 and R2 are time constant errors and are generally very small (smaller than the markers). The errors in the ^1^H-^15^N steady state NOE are a function of the S/N in the acquired spectra.

The structure of m_3_-HwTx-IV was determined using multidimensional heteronuclear NMR spectroscopy. NMR has been the dominant approach for determining the structure of peptides smaller than 5 kDa, including disulfide-rich venom peptides [22, 30]. Employing bacterial expression enabled us to produce uniformly ^15^N/^13^C labelled m_3_-HwTx-IV. Two-dimensional (2D) ^1^H-^15^N HSQC (Fig 2A) and 3D HNCACB, CBCA(CO)NH, and HNCO experiments were used to assign the backbone resonances of m_3_-HwTx-IV. A 4D HCC(CO)NH-TOCSY experiment, which has the advantage of providing sidechain ^1^H-^13^C correlations [25], was used to provide side-chain assignments. Ultimately, structure calculations were based on interproton distances derived from analysis of 3D ^15^N-edited and ^13^C-edited HSQC NOESY spectra and backbone dihedral-angle restraints derived from analysis of ^1^H, ^13^C, and ^15^N chemical shifts.

All non-exchanging and slow exchanging hydrogen atoms were assigned, as well as all protonated carbon and nitrogen atoms (BMRB-ID: 30160). These assignments were supplemented with assignments of all C’ atoms from the HNCO experiment, which excludes assignments from residues preceding the prolines and the C-terminal residue (N10, I35). The backbone chemical shifts were used to derive 46 dihedral angle predictions (23 ϕ, 23 ψ) by TALOS-N [31]. A total of 1575 NOEs were manually picked from the three NOESY spectra (^15^N, ^13^C aromatic, ^13^C aliphatic) and 1537 (98%) automatically assigned by CYANA [29]. These were translated to 790 unique distance limits. The disulfide bond connectivities were derived from unambiguous NOE assignments across the disulfide bond [32] and introduced into the structure calculations as three upper and lower distance limits across the disulfide bond (S_γ_-C_β_’, C_β_-S_γ_’, S_γ_-S_γ_’).

The structure calculations generated an ensemble of 20 structures (see Table 1 for statistics, PDB-ID: 5T3M). The ensemble was well defined with low backbone and sidechain RMSDs (0.02 Å and 0.48 Å respectively for residues 2–34). This compares favourably with the previously determined structure of the WT peptide (PDB ID 1MB6; RMSDs of 0.34 Å and 1.01 Å for the backbone and sidechain atoms of residues 2–34 [19]). The m_3_-HwTx-IV structure shows that the aromatic ring of W30 is directly above the H_β_ atom of T28, explaining the unusual upfield chemical shift of this atom at 2.48 ppm (compared with the random coil value of 4.16 ppm).

**Table 1:** NMR structure statistics^a^.

Experimental restraints		
Inter-proton distance restraints		
*Intra-residue*		199
*Sequential*		221
*Medium-range (i–j < 5)*		124
*Long-range (i–j > 5)*		246
Dihedral-angle restraints		46 (ϕ = 23, ψ = 23)
Disulfide-bond restraints		9
Total number of restraints per residue		24.4
RMSD from mean coordinate structure (Å)^b^		
Backbone atoms (residues 2–34)		0.02 ± 0.01
All heavy atoms (residues 2–34)		0.48 ± 0.07
Stereochemical quality^c^		
Residues in most favoured Ramachandran region (%)		90.91
Ramachandran outliers (%)		0 ± 0
Unfavourable sidechain rotamers (%)		7.97 ± 0.03
Clashscore, all atoms^b^		7.27 ± 0.00
Overall MolProbity score		2.58 ± 0.14 (~50^th^ percentile)

^a^All statistics are given as mean ± SD.

^b^Mean r.m.s. deviation calculated over the entire ensemble of 20 structures.

^c^As reported by Molprobity %.

### Dynamics of m_3_-HwTx-IV

Heteronuclear spin relaxation is a powerful technique to probe the overall and internal dynamics of macromolecules [33]. In order to study the dynamics of m_3_-HwTx-IV, we determined the ^15^N longitudinal (R1) and transverse (R2) relaxation rates and ^1^H-^15^N heteronuclear NOEs. The R1 and ^1^H-^15^N heteronuclear NOE values provide information about fast time-scale motions in the range of ns to ps while the R2 values probe slow time-scale motions in the range of ms to μs (Fig 3). The experimentally determined R1 values were found to be largely constant along the polypeptide chain with an average value of 2.25 ± 0.01 s^–1^. Residues at the N- and C-termini (C2 and I35) had shorter R1 values indicative of faster motions. In contrast to R1, the R2 values varied considerably along the polypeptide chain, ranging from 1.79 ± 0.01 to 7.65 ± 0.02 s^–1^ with an average value of 3.83 ± 0.02 s^–1^. Residues in the S25–T28 stretch, displayed higher or lower values than the average R2, suggestive of conformational exchange. The ^1^H-^15^N heteronuclear NOE was almost constant along the polypeptide chain (0.59 ± 0.03) with the exception of the flexible C-terminal residue (I35), which has a negative NOE, indicating a high level of disorder.

### Na_V_1 subtype selectivity of m_3_-HwTx-IV

**Fig 4:**
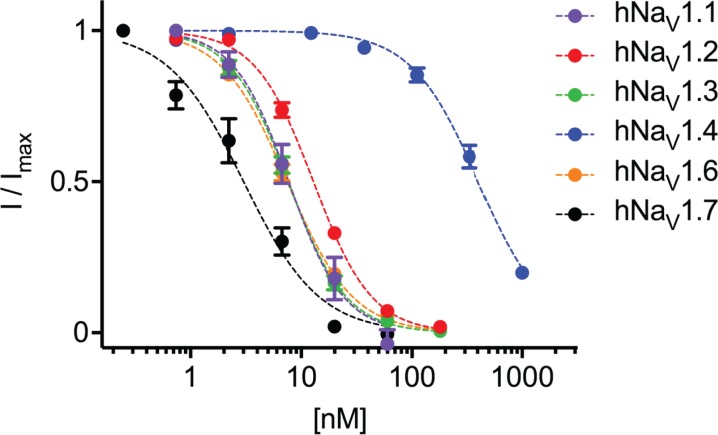
Potency and subtype selectivity of m_3_-HwTx-IV. Inhibition of hNa_V_1.1 –hNa_V_1.7 heterologously expressed in HEK293 cells (for hNa_V_1.1 –hNa_V_1.4) or CHO cells (for hNa_V_1.6 -hNa_V_1.8). Data points are mean ± SEM of three independent cells (*n* = 3) for hNa_V_1.3, four independent cells (*n* = 4) for hNa_V_1.1, hNa_V_1.4 and hNa_V_1.7, six independent cells (*n* = 6) for hNa_V_1.2 and of seven independent cells (*n* = 7) for hNa_V_1.6. Currents were recorded using automated patch-clamp electrophysiology (QPatch 16X). Fitting of the Hill equation to the data yielded IC_50_ values of (in nM) 3.3 ± 1.1, 6.8 ± 1.5, 7.2 ± 1.6, 8.4 ± 1.8, 11.9 ± 2.2 and 369 ± 196 for hNa_V_1.7, hNa_V_1.6 and hNa_V_1.3, hNa_V_1.1, hNa_V_1.2 and hNa_V_1.4, respectively. hNa_V_1.5 and hNa_V_1.8 were insensitive to the toxins up to 1 μM.

The full selectivity profile of m_3_-HwTx-IV at all human Na_V_ channel subtypes (hNa_V_1.1 –hNa_V_1.8) stably expressed in HEK293 (hNa_V_1.1 –hNa_V_1.5) and CHO (hNa_V_1.6 –hNa_V_1.8) cells was measured using whole-cell patch-clamp electrophysiology; fitting of the concentration-response data yielded IC_50_ values of: hNa_V_1.1, 8.4 ± 1.8 nM; hNa_V_1.2, 11.9 ± 2.2 nM; hNa_V_1.3, 7.2 ± 1.6 nM; hNa_V_1.4, 369 ± 196 nM; hNa_V_1.5, >1 μM; hNa_V_1.6, 6.8 ± 1.5 nM; hNa_V_1.7, 3.3 ± 1.1 nM; hNa_V_1.8, >1 μM (Fig 4 and Table 2), indicating that the neuronal Na_V_ isoforms 1.1, 1.2, 1.3, 1.6 and 1.7 are sensitive to the peptide whilst isoforms 1.4, 1.5 and 1.8 are not. The selectivity profile matched that of the parent (HwTx-IV) peptide, with the exception that activity against the Na_V_1.1, Na_V_1.6 and Na_V_1.8 had previously not been measured. The effects of m_3_-HwTx-IV on Na_V_ channel fast inactivation at 0 mV are shown in S1 Fig for the toxin sensitive channels (tested at ~ IC_50_ concentrations, expect for Na_V_1.4 which was assessed at 1 μM). m_3_-HwTx-IV had no substantial effect on the time constant of fast inactivation (τ) of any of the channels tested (S1 Fig).

**Table 2:** Potency of m_3_-HwTx-IV on hNa_V_1.1–1.8 stably expressed in HEK239 or CHO cells using whole-cell patch-clamp electrophysiology in QPatch and heterologously expressed in HEK293 cells assessed using FLIPR membrane potential assay. Data are presented as IC_50_ mean ± SD.

Subtype	IC_50_ (nM) QPatch	IC_50_ (μM) FLIPR
hNa_V_1.1	8.4 ± 1.8 (*n* = 4)	1.1 ± 0.7
hNa_V_1.2	11.9 ± 2.2 (*n* = 6)	0.54 ± 0.4
hNa_V_1.3	7.2 ± 1.6 (*n* = 3)	1.4 ± 0.6
hNa_V_1.4	369 ± 196 (*n* = 6)	>30
hNa_V_1.5	>1000 (*n* = 5)	>30
hNa_V_1.6	6.8 ± 1.5 (*n* = 7)	0.60 ± 0.6
hNa_V_1.7	3.3 ± 1.1 (*n* = 4)	5.1 ± 4
hNa_V_1.8	> 1000 (*n* = 11)	>30

In parallel, the full selectivity profile of m_3_-HwTx-IV at all hNa_V_1 channel subtypes heterologously expressed in HEK293 cells was measured using a FLIPR membrane potential assay, which yielded IC_50_ values of: hNa_V_1.1, 1.1 ± 0.7 μM; hNa_V_1.2, 0.54 ± 0.4 μM; hNa_V_1.3, 1.4 ± 0.6 μM; hNa_V_1.4, >30 μM; hNa_V_1.5, >30 μM; hNa_V_1.6, 0.60 ± 0.6 μM; hNa_V_1.7, 5.1 ± 4 μM; hNa_V_1.8, >30 μM (Table 2), supporting the selectivity data obtained using whole-cell patch-clamp electrophysiology.

### *In vivo* activity of m_3_-HwTx-IV

**Fig 5:**
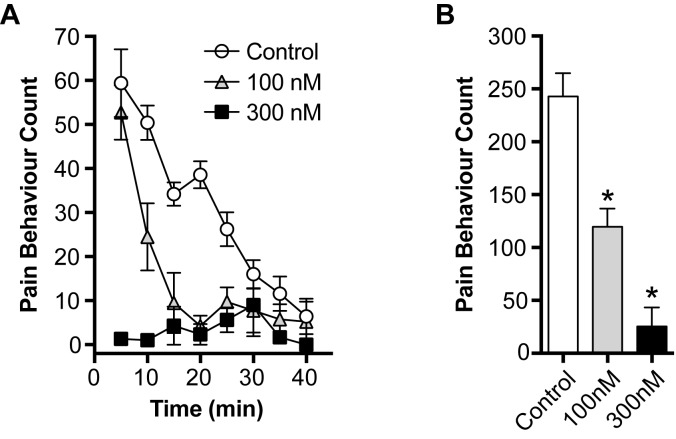
Dose-dependent suppression of OD1-induced spontaneous pain by m_3_-HwTx-IV. **(A)** Effect of co-administration of m_3_-HwTx-IV (i.pl.; 100 nM, 300 nM) on the time course of spontaneous pain behaviours elicited by intraplantar injection of the Na_V_1.7 activator OD1 in mice. **(B)** Effect of m_3_-HwTx-IV (i.pl; 100 nM, 300 nM) on total pain behaviours (0–40 min) induced by OD1 in mice. Data are presented as mean ± SEM, *n* = 3–5 mice per group. Statistical significance was determined using one-way ANOVA with Dunnett’s post-test, *P < 0.05 compared to control.

The on-target effect of m_3_-HwTx-IV at Na_V_1.7 was examined *in vivo* via behavioural assessment in a recently described Na_V_1.7 target engagement model [21]. m_3_-HwTx-IV significantly attenuated pain behaviours induced by local injection of the Na_V_1.7-selective activator OD1 [34] into the hind paw of the mouse model of Na_V_1.7-mediated pain (pain behaviour count: control (n = 5), 243 ± 22; m_3_-HwTx-IV (100 nM; n = 4), 120 ± 17; m_3_-HwTx-IV (300 nM; n = 3), 25 ± 18; *P* < 0.05; Fig 5), confirming on-target activity at Na_V_1.7.

## Discussion

### Structure of HwTx-IV and mutants

m_3_-HwTx-IV was developed by Revell et al. [14] to improve potency of the parent compound (HwTx-IV) at Na_V_1.7. Here we determined whether the improved activity affected the structure or selectivity of HwTx-IV. The structure of WT HwTx-IV was previously solved by two independent groups (PDB IDs 1mb6 and 2m4x for the original and more recent HwTx-IV structures, respectively). Notably, when these two structures are aligned over the backbone atoms in the structured region (residues 5–33), the RMSD was found to be 1.84 Å, which is remarkably high (i.e., indicative of significant differences), considering that both structures were solved using primarily 2D homonuclear NOESY data.

To structurally characterise m_3_-HwTx-IV we used a recombinant expression method [22] to isotope label the peptide with ^13^C and ^15^N, which allowed application of high-resolution, heteronuclear 3D/4D NMR methods as well as ^15^N relaxation measurments. The structure obtained here using heteronuclear NMR aligns well with the original native peptide structure, 1mb6, with an RMSD of 0.7 Å over the backbone atoms (5–33), but it aligns poorly with the more recent 2m4x structure, with an RMSD of 1.75 Å over the same range of backbone atoms. The large difference between our structure and the more recent of the two structures of the WT peptide is apparent in 3 of the 4 loops of the peptide as well as the termini. It is unclear why the 2m4x structure deviates to such an extent from the other two structures. We note, however, that for the 1mb6 structure the authors recorded separate 2D NOESY spectra in H_2_O and D_2_O and it may be that this approach provided additional ^1^H NOEs from Hα atoms in the β-sheet regions which are often obscured in 2D NOESY spectra by the strong H_2_O signal. In our 3D filtered NOESY data these NOEs are obscured along the direct dimension but can often be assigned along the indirect dimension. Given the discrepancy, and the additional experimental data used in the 1mb6 structure, we have below only made comparisons to 1mb6.

### Structural consequences of mutations

**Fig 6:**
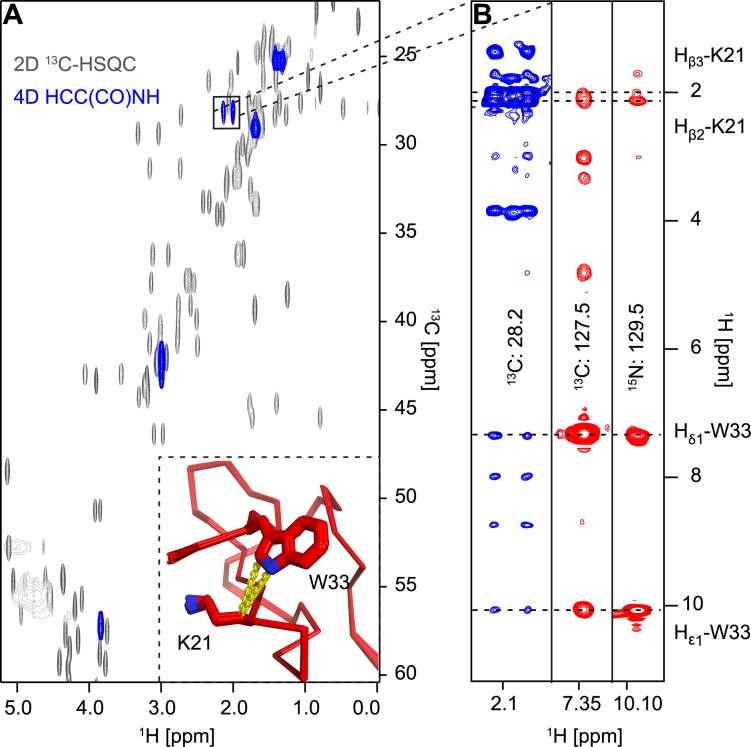
Interaction of K21 and W33 sidechains. **(A)** Overlay of the relevant plane from the 4D HCC(CO)NH spectrum and the ^1^H-^13^C projection of the ^13^C-HSQC-NOESY spectrum, indicating the unique chemical shifts of the H_β_ protons of K21. The inset shows the proximity of the β atoms of K21 to the H_δ1_ and H_ε1_ atoms of W33 in the NMR structure of m_3_-HwTx-IV. **(B)** Strip plot of the ^13^C-HSQC-NOESY (aliphatic region left, aromatic region middle) and ^15^N-HSQC-NOESY spectra (right) showing NOEs between the β atoms of K21 and the H_δ1_ and H_ε1_ atoms of W33 and vice versa.

In the m_3_-HwTx-IV structure the position of W33 with respect to K21 is well defined, whereas in the WT structure the aromatic ring of Y33 is poorly defined and predominantly positioned facing the sidechains of K32 and F6. In our structure there are clear NOEs between the sidechain H_β_ protons of K21 and the H_δ_1 and H_ε_1 protons of W33 in the 3D ^13^C aromatic NOESY, 3D ^13^C aliphatic NOESY and the 3D ^15^N NOESY spectra. The chemical shifts of these atoms are unique in the 3D NOESY spectra and the NOE assignments are therefore unambiguous, strongly supporting the alternative sidechain conformation in our model (Fig 6).

The NMR relaxation studies revealed no evidence of structural or conformational flexibility in the C-terminal tail, except for the last residue, which appears less ordered. It is therefore unclear wether the W33/K21 interactions are not present in the WT peptide or whether these interactions were simply not resolved in the previous homonuclear NMR studies. We note that in the 2D NOESY data the H_β_ atoms of K21 are in a crowded region, which may have obscured NOEs in the 1mb6 data. It had previously been speculated that cation-π interactions may serve to reduce the loss in energy upon binding due to the desolvation of lysine residues. Here, the conformation of these two residues would not provide such stabilisation, as the charged NH_3_^+^ group of the lysine is instead proximal to the carboxyl-terminus of the peptide.

The other residues that were mutated in m_3_-HwTx-IV are two glutamic acid residues in the N-terminal section of the peptide. Curiously the E4 residue appears to form a salt bridge with K7 in the WT toxin, and it might be expected that this charge pairing stabilises the fold of the peptide. However, in our structure K7 assumes a nearly identical position as in the WT peptide whilst there are no major changes to the conformation of loop 1. Thus, the improved Na_V_1.7 activity from mutating these acidic residues to Gly likely results from removal of unfavourable interactions with either VSD_II_ of Na_V_1.7 or the surrounding membrane. Indeed, previous work has shown that loop 1 residues are important in lipid binding and it is possible that the acidic residues either interact unfavourably with anionic components of the membrane leaflet or reduce the permeability of the peptide in the lipid bilayer [35].

The C-terminus of HwTx-IV is amidated and previous structure/activity studies have shown that removal of this amidation causes a 20–50 fold reduction in activity at Na_V_1.7 [14, 36]. Since m_3_-HwTx-IV was produced recombinantly, the C-terminus is not amidated and we would expect the activity of the peptide to be less than that of the amidated peptide. Indeed we found that the recombinant peptide inhibited Na_V_1.7 with an IC_50_ of 3.3 nM, which is 8-fold higher than that previously reported for the synthetic amidated version but still more potent than WT HwTx-IV (IC_50_ 26 nM). Thus, m_3_-HwTx-IV is one of the most potent recombinantly produced Na_V_1.7 blockers reported, and in contrast to ProTx-II, it does not require refolding [37].

### Dynamics of the pharmacophore region

The Na_V_ pharmacophore in this class of peptides has been extensively characterised and it is clear that the W30/K32 pair are the most functionally critical residues [14, 20]. In our structure these residues assume a conformation similar to that in WT peptide, and we conclude that the three mutations do not affect the overall configuration of these critical residues. Instead, our relaxation data show clear evidence of conformational mobility in loop 4 residues including W30. Remarkably, we find that the largest discrepancy between our mutant structure and the WT structure is in this loop, and when this loop is omitted in the structural alignment the RMSD falls from 0.7 Å to 0.4 Å. This suggests that this loop is flexible and that the difference between the structures may simply reflect different conformational states of loop 4 (see also Fig 2B). Given that this dynamic region coincides with the active site of the peptide we speculate that this mobility provides additional conformational states that may stabilise binding of the peptide to VSD_II_. These data also suggest that the absolute configuration of the pharmacophore residues in the solution structure of HwTx-IV may differ from that of the channel-bound peptide. Should this be the case, the solution structures may not be suitable design templates for development of peptide mimetics or small molecules inhibitors, unless sufficient flexibility is introduced with respect to the position of these key functional residues.

### Subtype selectivity

The binding site of HwTx-IV was characterised in detail by Xiao et al. [16]. The authors found that HwTx-IV inhibits rat (r) Na_V_1.2 and rNa_V_1.3 as well as human Na_V_1.7, but is far less potent on rNa_V_1.4 and rNa_V_1.5. Based on these results and careful mutagenesis work, Xiao et al. were able to identify residues on Na_V_s that conferred selectivity [17]. They showed that the peptide binds to VSD_II_ and that one residue on the S1-S2 loop (E753), together with four residues on the S3-S4 loop (E811, L814, D816, E819), are particularly important for peptide binding. This recognition motif “EELDE” is conserved in Na_V_1.6, whilst only differing in a D-to-N change (EELNE) in Na_V_1.1, Na_V_1.2, Na_V_1.3. In contrast, Na_V_1.4 and Na_V_1.5 contain two changes (EELNQ and EELRS respectively), and Na_V_1.8 contains three changes (EEVKG) with Na_V_1.9 having the biggest difference with four changes (EDVQR).

From the above analysis it should therefore be expected that Na_V_1.6 should be equally sensitive to m_3_-HwTx-IV, and since Na_V_1.2 is sensitive to the toxin, we would expect Na_V_1.1, which has the same recognition sequence, to also be m_3_-HwTx-IV sensitive. Finally, based on the sequence analysis, Na_V_1.8 and Na_V_1.9 are least likely to be sensitive to the toxin. Using a FLIPR membrane potential assay we compared subtype selectivity profiles across Na_V_ isoforms and as predicted by the work in the Cummins group [17, 38] the previously uncharacterised hNa_V_1.6 and hNa_V_1.1 are indeed sensitive to the toxin whilst hNa_V_1.8 is, again as predicted, insensitive to the toxin. To further characterise the selectivity of m_3_-HwTx-IV we measured its activity against Na_V_1.1–1.8 using whole cell patch-clamp electrophysiology. We found that the peptide is very potent on Na_V_1.1–1.3, Na_V_1.6 and Na_V_1.7. Thus, it appears that the D-to-N mutation at position 816 is well tolerated. Comparing the activity on Na_V_1.4/5 and Na_V_1.1 we can conclude that final E-to-Q/S mutation is not tolerated and the presence of a negative charge is essential in this position. The full range of Na_V_ subtypes sensitive to HwTx-IV only differs from tetrodotoxin (TTX) in its activity on Na_V_1.4. This may prove useful as differential inhibition of sodium currents by HwTx-IV and TTX can be attributed to Na_V_1.4.

Our results show that, despite claims of improved selectivity and potency, the subtype selectivity of m_3_-HwTx-IV is not significantly altered compared with native HwTx-IV. From the sequence analysis our data would support a model where the peptide binds to residues within the cavity formed between the four helices of the VSD_II_ [39]. This model would suggest that there is a large interface between the peptide and the channel, indicating that it should in theory be possible to develop subtype selective peptides. Current peptide engineering efforts, however, rely on exhaustive mutagenesis of the peptide and have unfortunately shown limited success, with the common observation that improvements in affinity against one of the sensitive subtypes often leads to a correlated increase in affinity against other subtypes [40]. To overcome these challenges further structural details of the VSD_II_ in its peptide bound form are required to enable rational peptide design.

### *In vivo* activity

The *in vivo* activity of HwTx-IV was previously reported in rat models of formalin-induced pain and spared nerve injury, where it blocked pain behaviours at doses between 25 and 200 μg/kg [41]. This exquisite *in vivo* potency may at least in part be due to the strain of animals used, as morphine also was effective at doses at least 20 times lower than those usually required to achieve analgesia in the same models [42–45]. Given the poor selectivity of HwTx-IV, in particular over Na_V_1.6, we used a recently developed Na_V_1.7 target engagement assay to assess the biological activity of recombinant m3-HwTx-IV following local intraplantar injection, as systemic administration of peptides with activity at Na_V_1.6 typically leads to adverse side-effects. Intraplantar injection of OD1 at the concentration used here evokes spontaneous pain behaviours that are reduced by ~70% in Na_V_1.7 knockout mice or after treatment with the selective Na_V_1.7 inhibitor PF-04856264, with residual pain responses likely attributable to Na_V_1.6 [21]. Accordingly, the observed *in vivo* effects of m3-HwTx-IV are entirely consistent with our pharmacological characterization of m3-HwTx-IV showing potent effects at Na_V_1.6 and Na_V_1.7, and indicate target engagement of Na_V_1.7 and to lesser extent Na_V_1.6 on peripheral nerve terminals *in vivo*. The *in vivo* effects of m3-HwTx-IV were only assessed in male mice, as sex-related differences in Na_V_1.7-mediated pain are not expected [46].

## Conclusions

We report here an efficient method of recombinant production of a highly potent Na_V_1.7 inhibitor, m_3_-HwTx-IV. Our high-resolution solution-state NMR structure revealed that the mutations introduced into HwTx-IV do not alter the structure of the peptide. Thus, with perhaps the exception of the Y33W mutation, the gain in potency for the mutant peptide appears to be due to removal of unfavourable contacts from the acidic moieties (E1G, E4G). Furthermore, we provide the first NMR relaxation studies of this family of sodium channel inhibitors, which revealed significant conformational flexibility in the critical pharmacophore region. Detailed functional studies showed that the subtype selectivity of m_3_-HwTx-IV differs from TTX only at Na_V_1.4, which was not sensitive to m_3_-HwTx-IV. Finally, we found that m_3_-HwTx-IV provides analgesia in a mouse model of Na_V_1.7-mediated pain. While recombinant m_3_-HwTx-IV is likely to be a useful pharmacological tool for structural and pharmacological studies, its off-target activity makes it a poor clinical candidate. Further structural details of the peptide-channel complex are likely required to enable design of HwTx-IV analogues with improved selectivity profiles.

## Supporting information

S1 Fig m_3_-HwTx-IV has no effect on Na_V_ channel fast inactivation when tested near the IC_50_ value. Na^+^ currents were elicited by a 20 ms depolarising pulse to 0 mV from a 200 ms pre-pulse at -120 mV. Holding potential was -80 mV. The time constant of fast inactivation (τ) was calculated from a fit of the inactivation phase to a mono exponential equation and is presented as mean +/- SEM (n = 3 repetitions).
(DOCX)
