# Supplementary material for: The structure, dynamics and selectivity profile of a NaV1.7 potency-optimised huwentoxin-IV variant
Source: PLoS One. 2017 Mar 16;12(3):e0173551. doi: 10.1371/journal.pone.0173551 (PMC5354290; doi:10.1371/journal.pone.0173551)
Supplement: S1 Fig — Na+ currents were elicited by a 20 ms depolarising pulse to 0 mV from a 200 ms pre-pulse at -120 mV. Holding potential was -80 mV. The time constant of fast inactivation (τ) was calculated from a fit of the inactivation phase to a mono exponential equation and is presented as mean +/- SEM (n = 3 repetitions). (DOCX) [file pone.0173551.s001.docx]

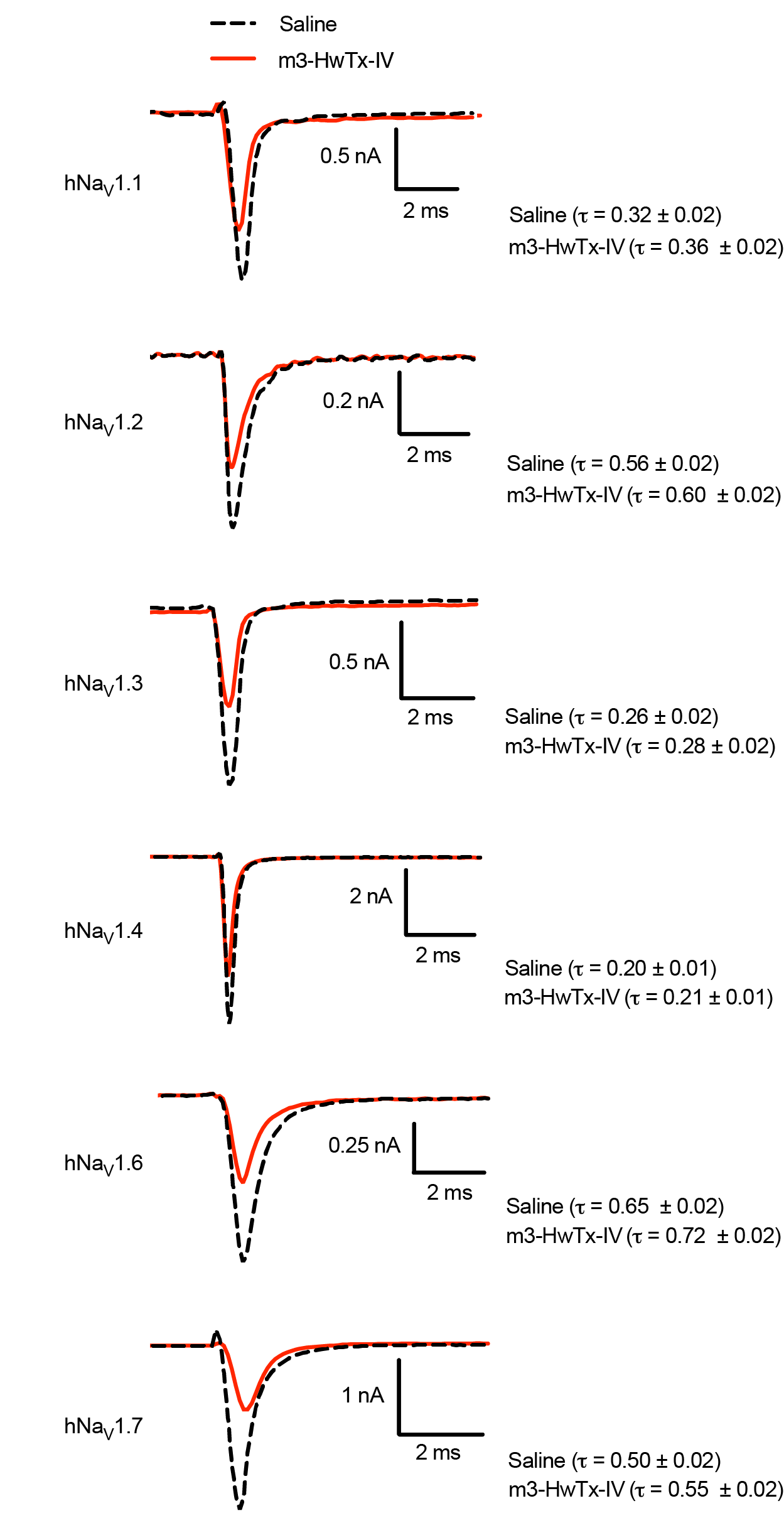


**Supplemental Figure S1. m_3_-HwTx-IV has no effect on Na_V_ channel fast inactivation when tested near the IC_50_ value.** Na^+^ currents were elicited by a 20 ms depolarising pulse to 0 mV from a 200 ms pre-pulse at -120 mV. Holding potential was -80 mV. The time constant of fast inactivation (τ) was calculated from a fit of the inactivation phase to a mono exponential equation and is presented as mean +/- SEM (n=3 repetitions).
